# Spatial variability of low frequency brain signal differentiates brain states

**DOI:** 10.1371/journal.pone.0242330

**Published:** 2020-11-12

**Authors:** Yifeng Wang, Yujia Ao, Qi Yang, Yang Liu, Yujie Ouyang, Xiujuan Jing, Yajing Pang, Qian Cui, Huafu Chen

**Affiliations:** 1 Institute of Brain and Psychological Sciences, Sichuan Normal University, Chengdu, China; 2 The Clinical Hospital of Chengdu Brain Science Institute, MOE Key Lab for Neuroinformation, School of Life Science and Technology, University of Electronic Science and Technology of China, Chengdu, China; 3 Tianfu College of Southwestern University of Finance and Economics, Chengdu, China; 4 School of Public Affairs and Administration, University of Electronic Science and Technology of China, Chengdu, China; School of Psychology, CHINA

## Abstract

Temporal variability of the neural signal has been demonstrated to be closely related to healthy brain function. Meanwhile, the evolving brain functions are supported by dynamic relationships among brain regions. We hypothesized that the spatial variability of brain signal might provide important information about brain function. Here we used the spatial sample entropy (SSE) to investigate the spatial variability of neuroimaging signal during a steady-state presented face detection task. Lower SSE was found during task state than during resting state, associating with more repetitive functional interactions between brain regions. The standard deviation (SD) of SSE during the task was negatively related to the SD of reaction time, suggesting that the spatial pattern of neural activity is reorganized according to particular cognitive function and supporting the previous theory that greater variability is associated with better task performance. These results were replicated with reordered data, implying the reliability of SSE in measuring the spatial organization of neural activity. Overall, the present study extends the research scope of brain signal variability from the temporal dimension to the spatial dimension, improving our understanding of the spatiotemporal characteristics of brain activities and the theory of brain signal variability.

## Introduction

The human brain is a complex adaptive system with significant spatiotemporal variability. Beyond stochastic noise, temporal variability has been suggested to reflect the dynamic range of brain function, providing kinetic energy for the brain to achieve various potential functional states [1]. Greater temporal variability is usually associated with better cognitive performance [2] and healthier brain state [3]. By contrast, another group of studies have observed associations between greater temporal variability and poorer cognitive performance [4–6], suggesting a complicated relationship among the temporal variability, cognitive performance, age, and other factors. These studies about brain signal variability, compared with investigations about mean brain signal, have uncovered some significant findings of the brain behaving as a complex system [7,8].

Like the local temporal variability, the inter-regional variability could reflect specific brain states [9]. For instance, reliable distribution of low frequency amplitude across brain regions has been observed during resting state [10,11] and has been shown to change during task state [12,13]. Particular spatial patterns are closely associated with specific cognitive functions [12,14] and have been utilized to predict brain states [15,16]. Obviously, a specific cognitive processing is associated with a particular temporal pattern and a particular spatial pattern of neural activities. Therefore, we hypothesize that besides temporal variability, the spatial variability of brain signal may also provide important information about brain function.

Sample entropy (SE) has been utilized to evaluate the variability or self-resemblance of a time series in functional magnetic resonance imaging (fMRI), electroencephalogram (EEG), magnetoencephalogram (MEG), and other physiological signals [17–19]. It has been suggested to be a biomarker of health and adaptive capacity in disease and aging [20,21]. Compared to linear indices such as standard deviation and mean squared successive difference [12,22], the SE reflects both linear and nonlinear autocorrelations, providing more information about the underlying brain state [17,18]. Compared with other nonlinear indices such as Lyapunov exponent and approximate entropy, the SE has been found to be robust to occasional, very large or small noises, and relatively short data segments [23,24]. These characteristics enable SE to measure fMRI data with short data length. Recent fMRI studies have demonstrated that SE is effective in predicting aging [23], distinguishing brain networks [25], and representing cognitive functions [26]. Therefore, we adopted SE to evaluate the spatial variability of brain signal during distinctive brain states.

Low frequency steady-state brain response (lfSSBR) is a steady-state evoked potential (SSEP)-like phenomenon in the low frequency range (< 1 Hz) which is evoked by cognitive tasks or stimuli presented in a fixed frequency [27]. This paradigm has been widely used in fMRI studies [12,28–31]. Compared with transient brain activation measured by mean brain signal, the lfSSBR reflects brain signal variability during a stable brain state in a relatively long time course [28]. Furthermore, compared to transient brain activation, the lfSSBR has a much higher signal to noise ratio at the task frequency, thus can effectively detect some faint brain activities [28,30]. Energy rearrangement has been observed during lfSSBR, which changes the inter-regional relationship in widespread brain areas [12,32]. These characteristics make the steady-state task an ideal experimental paradigm to compare spatiotemporal variability during a particular task state and resting state.

In the present study, we highlighted the importance of spatial variability of neural activity in distinguishing brain states and put forward spatial SE (SSE) to evaluate the spatial variability of fMRI signal during task state and resting state. It has been suggested that spontaneous activity reflects the stochastic exploration of the high-dimensional space [33,34]. In contrast, the cognitive processing invoked by external stimuli transfers this space into functionally relevant subspaces [35], causing responses to exhibit lower dimensionality and lower variability [36]. Regular inter-regional relationship with low variability is essential to maintain the system at constrained states to improve cognitive performance [37]. According to these findings, we expected smaller SSE during task state than during resting state.

## Methods

Data in this study has been described in detail in a previous study and is therefore only briefly described here [12].

### Subjects

Thirty participants (age range: 18–27 years, mean ± SD = 22.41 ± 2.11 years; 15 females) were recruited for this study. All of them had normal or correct to normal vision, were right-handed, reported free from any medication, psychiatric, and neurological disorders. All the procedures used on human participants were in accordance and approved by the ethical standards of School of Life Science and Technology research committee and with the 1964 Helsinki declaration and its later amendments or comparable ethical standards. Written informed consent, approved by the research ethical committee of School of Life Science and Technology at University of Electronic Science and Technology of China (UESTC), was obtained from each subject before the beginning of the experiment.

### Task procedure

Participants were required to perform a face detection task by judging whether the face has a neutral expression (right thumb response) or happy expression (left thumb response) as accurately and fast as possible. Although there were only neutral faces in the paradigm, subjects were told that happy expression appeared no more than once to ensure that they paid attention during the entire task. All stimuli were selected from the Chinese Facial Affective Picture System. The values of valence, arousal, dominance, and attraction were 4.40 ± 0.60 (mean ± SD), 3.65 ± 0.54, 4.98 ± 0.35, and 4.19 ± 0.45, respectively. In each trial, the face was presented on the black background for 2 s and followed by a white crosshair of 18 s. Each trial lasted for 20 s, forming a fundamental frequency of 0.05 Hz. There were 31 trials in all, constituting a run of 620 s. The procedure was conducted with E-Prime 2.0 software (http://www.pstnet.com).

A resting scan lasted for 620 s. The order of resting scan and task scan was counterbalanced between subjects. Participants were asked to remain motionless, focus their eyes on a white crosshair, stay awake, and not think of anything in particular during the resting scan.

### Image data acquisition

The fMRI data were acquired using a 3.0T GE 750 scanner (General Electric, Waukesha, WI USA) at UESTC with the gradient-recalled echo-planar imaging (EPI) sequence. An 8-channel prototype quadrature birdcage head coil fitted with foam padding was applied to minimize the head motion. The imaging parameters were as follows: repetition time/echo time = 2000 ms/30 ms, 90° flip angle, 64 × 64 matrix, 22 cm field of view, 43 axial slices (3.2 mm slice thickness without gap).

### Image data preprocessing

Functional images were preprocessed using the Data Processing Assistant for Resting state fMRI (DPARSF 2.3) [38]. The first 10 volumes were discarded to ensure signal equilibrium, to allow evoked fluctuations to appear, and for the participants to familiarize themselves with the scanning environment [27]. The remaining 300 images were slice-time corrected, spatially aligned, spatially normalized to Montreal Neurological Institute (MNI) EPI template and resampled to 3 × 3 × 3 mm^3^ voxels. The images were spatially smoothed (8-mm FWHM Gaussian kernel). Friston 24 motion parameters [39], white matter signal and cerebrospinal fluid signal were further extracted and regressed out using the DPARSF software. The data of one participant was removed due to large head motion (translation >3 mm or rotation >3°) in any scan. Finally, following previous studies using the steady-state paradigm [12,27,29,30,40,41], band-pass filter was performed within a narrow frequency band of 0.0475–0.0525 Hz (the fundamental frequency of task) for both task state and resting state data. The narrow band filter was used because the task effect was primarily limited in the fundamental frequency of task compared with the resting state. Like those studies using the steady-state paradigm [12,31,40], both task and rest were analyzed the same way to obtain a comparable baseline of the task while eliminating noise interference from other frequency bands.

### Behavioral data analysis

The accuracy and reaction time (RT) of behavioral performance were calculated for each subject.

### Spatiotemporal SE calculation

The preprocessed data were divided into 246 regions using the Brainnetome Atlas [42,43]. At each time point, the blood oxygen level dependent (BOLD) signals of all voxels within each region were averaged. The values of 246 brain regions were arranged in their labels’ order. BOLD signal was extracted for each subject, forming a matrix of 246 regions × 300 time points. Instantaneous amplitude of each region across 300 time points was calculated by Hilbert transformation. The SSE at each time point was calculated based on instantaneous amplitude of each region according to Eq (1):

$$SE(m,r,N)=-\log\frac{C^{m+1}(r)}{C^{m}(r)}$$ (1)

where m is the pattern length, r (similarity factor) which represents a proportion of the standard deviation (SD) of the signal series is a distance threshold, N is the length of the signal sequence (here N = 246), *C^m^(r)* measures the average likelihood of m-length patterns in a signal series. Two patterns match if the distance is less than r. Prior studies have suggested that data length of 10^m^–20^m^ is reasonable to estimate SE [26]. Therefore, m = 1 and m = 2 were assessed for the data length of 246. Following prior studies [23,26], r = 0.05 to 0.50 were assessed. To demonstrate the tolerance of SSE to reordered data, the SSE was retested based on the signal with 246 values aligned in odd then even labels. Both task state and resting state were analyzed in the same way.

### Statistical analysis

SSEs were compared between task state and resting state using the permutation test. Because the amplitude of neural activities across brain regions is stable and regionally specific during resting state [10], the SSE under resting state would result in functionally meaningful baseline rather than “0” baseline for task state. Paired-samples t-test was conducted between SSEs at two randomly selected time points under the task state and resting state, respectively. This test was repeated for 10,000 times under each combination of m and r. The mean of 10,000 p values was deemed as a significant level. Following previous studies [23,26], reported results were based on parameters produced the largest difference between task state and resting state. These parameters were defined as the optimal parameters.

To test the difference between two states, the mean and SD of SSE of 300 time points were further compared between task state and resting state using paired-samples t-test. Furthermore, Pearson’s correlation was performed between the mean and SD of SSE and RT, respectively.

## Results

### The optimal parameters of SSE

**Fig 1:**
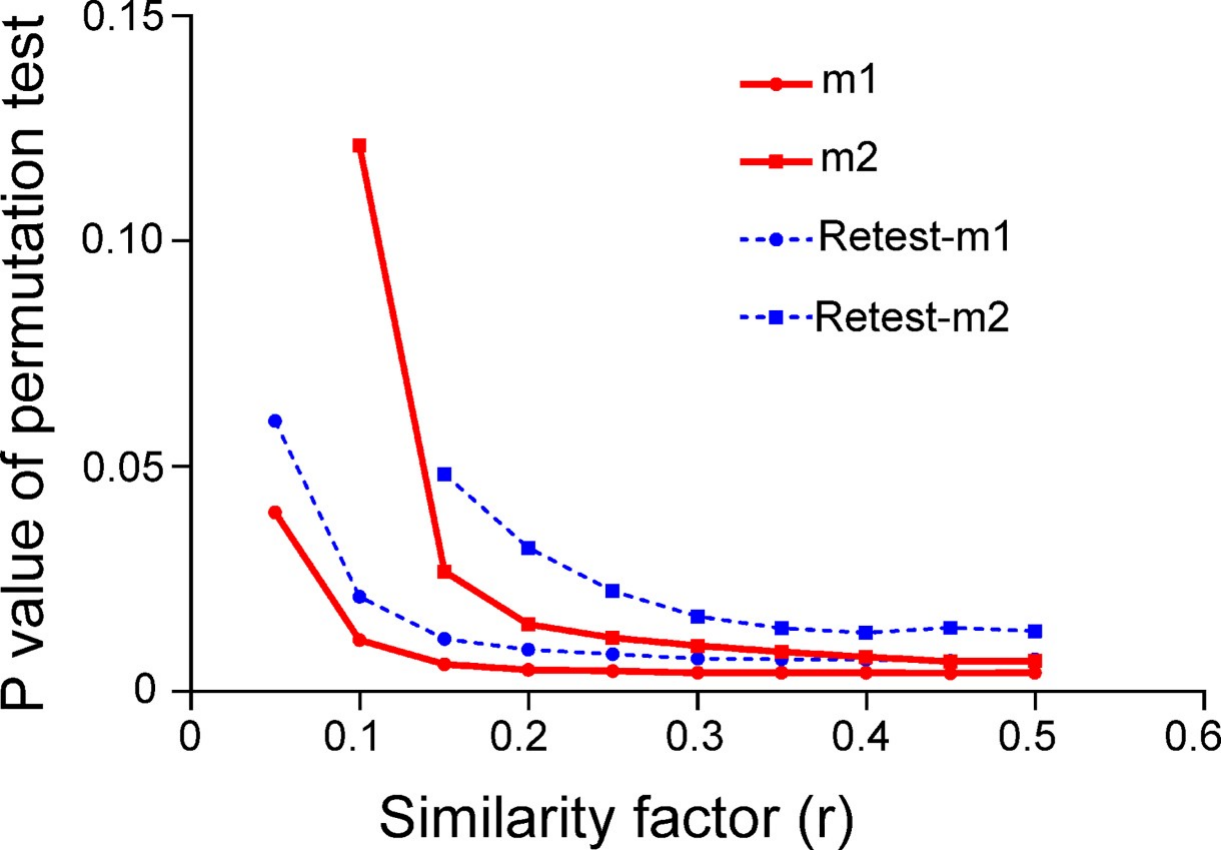
Parameter optimization of spatial sample entropy. The mean p values of permutation test between task state and resting state are illuminated. Some results are not shown because the p values are infinite. The largest discrepancy between task state and resting state appears at m = 1 and r = 0.45 for both test and retest data. These differences were significant after Bonferroni correction (p < 0.05).

In the preliminary calculation, differences between task state and resting state were always significant for different combinations of m and r as long as r > 0.1, suggesting the robustness of current results. The largest difference occurred at m = 1 and r = 0.45. These parameters were compatible with previous fMRI studies [23,26], ensuring the rationality of the current results. These parameters are replicated by test-retest (see Fig 1) and are deemed as the optimal parameters. Subsequent results are based on these parameters.

### SSE is lower in the task state than in the resting state

**Fig 2:**
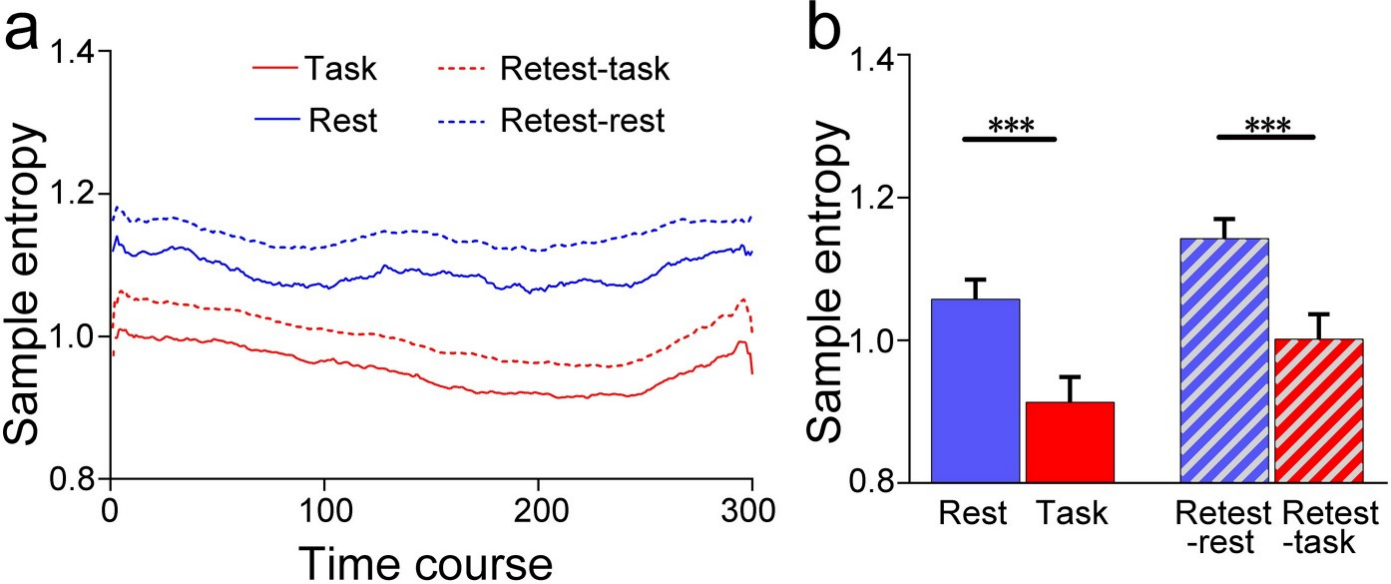
The group mean of spatial sample entropy. Panel A shows the spatial sample entropy along with 300-time points. Panel B shows the mean spatial sample entropy across time. Error bar indicates 95% confidence interval.

As shown in Fig 2, the mean SSE of 300 time points is lower during task state than during resting state [t (28) = 6.00, p = 1.82e-6 for test and t (28) = 6.03, p = 1.68e-6 for retest]. Permutation test indicated a lower SSE during task state than during resting state (p = 0.0041 for test and p = 0.0068 for retest) across 300 time points, indicating that SSE can differentiate task state and resting state.

No statistical difference was found on the SD of SSE between task state and resting state [t (28) = 0.01, p = 0.99 for test and t (28) = 1.09, p = 0.28 for retest], indicating the stable temporal characteristics of SSE across brain states.

### Increased temporal variability of the SSE is associated with more stable task performance

The subjects judged face expressions with extremely high accuracy. There were only two incorrect responses out of 870 trials in all 29 subjects. Therefore, the accuracy was not involved in further analyses. RTs in these two trials were replaced with the mean RT of that subject. At the group level, the mean RT was 624.67 ± 107.47 (mean ± SD) ms, ranging from 344.32 to 875.43 ms. The SD of RT ranged from 50.88 to 184.80 ms.

**Fig 3:**
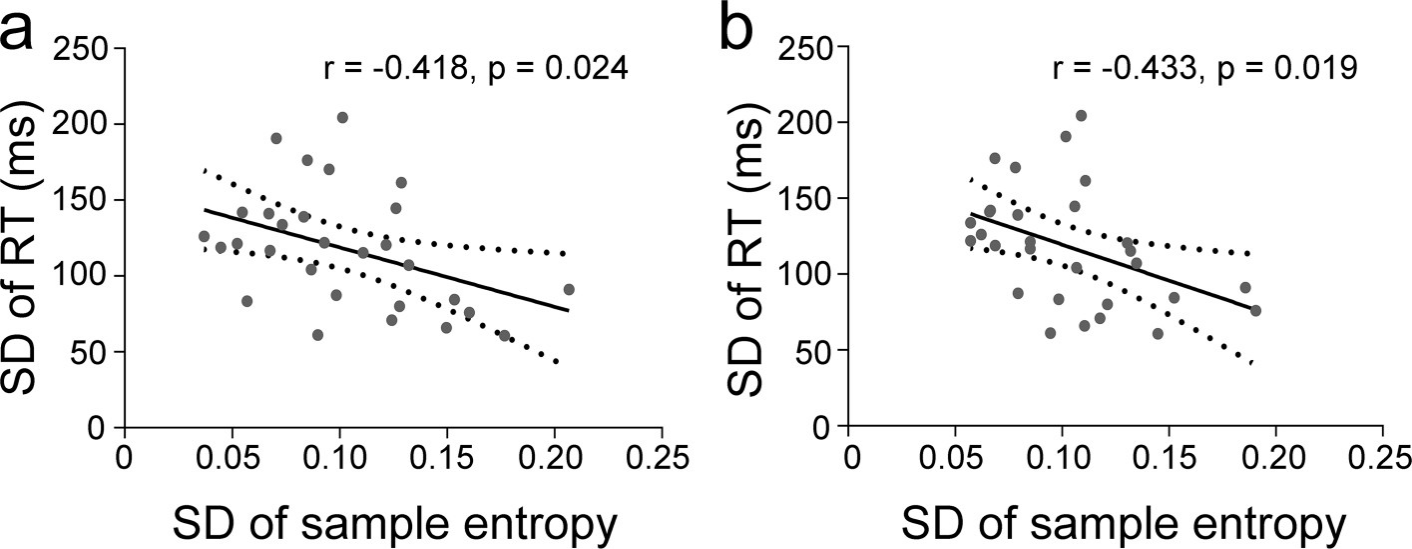
The negative correlation between the SD of spatial sample entropy and the SD of RT. Panel a and b show correlations in test and retest data, respectively. Dotted lines show 95% confidence intervals.

The negative correlation was observed between the SD of RT and SD of SSE during task state (see Fig 3), suggesting that larger temporal variability of SSE during task state was associated with more stable behavioral performance.

These results are replicated by test-retest study, suggesting the high reliability of SSE results and supporting that SSE is tolerant to short and reordered data to some extent [23,24].

## Discussion

This study put forward the spatial SE to measure the spatial patterns of different brain states and revealed reduced spatial variability of the human brain during a face detection task. We compared for the first time, spatiotemporal SEs between task state and resting state. According to the flexibility/stability hypothesis [2], reduced SSE indicates that neural system transfers from multiple potential states to a few stable states to facilitate the task. The negative correlation between the variabilities of SSE and RT extends the physiological signification of temporal variability of local neural activity, suggesting that the particular spatial pattern of neural activity is of importance for task performance.

Higher entropy means that the brain network produces a larger number of possible activity configurations that are not explained by the standard parametric analysis of the noise [37]. Using the lfSSBR paradigm, we revealed decreased SSE during face detection, suggesting smaller number of possible spatial interactions during the task. Reduced SSE during task state is in accord with our hypothesis and previous findings that brain networks during cognition, compared with resting state, reorganize from the metastable state to the more stable state [8,44]. Of note, the temporal variability was increased from resting state to task state in most previous studies, including ours [2,27,29,41,45]. The opposite patterns of task modulated temporal variability and spatial variability suggest that cognitive tasks may reorganize brain signals in the temporal dimension and spatial dimension simultaneously. Although the temporal variability was increased, the mean temporal signal was both increased and decreased in different regions during the task state [45,46], which may meanwhile reorganize the spatial relationship. It has been suggested that if the task requests a sinusoidal signal output, the complexity would be decreased; whereas the task asks a constant signal output, the complexity would be increased [47,48]. The entrainment mechanism of lfSSBR could make brain signals in core and extended face areas be sinusoidal [29,49], further reducing SSE by enhancing the regularity of the signal. The SSE at each time point is lower during task state than during resting state, suggesting that the spatial pattern for the amplitude of spontaneous neural activity is more variegated than that of task-evoked neural activity at each time point. The great difference between task state and resting state makes SSE a stable proxy of brain state [33].

Although the SSE during task state is lower than that during resting state, their temporal variabilities were comparable. In other words, although SSE varies from state to state, it is stable for the same state of the same person. The stability of SSE enables it to reflect individual differences such as the reaction speed and anxiety level [43]. Three reasons may cause the similar temporal variabilities between task state and resting state. First, there is no task switch just like that in block experimental design; therefore, there is no switch between on and off brain states. Second, the brain state is stable during lfSSBR [27,28]. Third, the task state and resting state are associated with externally- and internally-oriented attention respectively, which have similar temporal characteristics [50].

The negative correlation between the SD of SSE and the SD of RT is in line with previous findings that larger temporal variability is associated with better behavioral performance [2,7,51]. Greater temporal variability may provide kinetic energy for brain function, enabling the brain to explore a variety of potential functional states. There is also a literature that shows increased variability is predictive of poorer cognitive performance, especially in lifespan samples [4–6]. This inconsistency suggests that age, task difficulty, and other factors such as the dopaminergic neurotransmission and brain regions can modulate the relationship between temporal variability and cognitive performance. Although there are many factors worth exploring, the significance of temporal variability has been verified in local and network levels [33,52,53]. Here we expand this brain-behavior relationship to the global spatial configuration of neural activity, suggesting that the temporal variability of brain signal is an effective proxy of brain function at multiple levels and the SSE is a powerful indicator of brain state.

Although the temporal variabilities of SSE were comparable between task and resting states, that during task state was associated with the efficiency of face detection, indicating the temporal variability of SSE is state-related and supporting the link between spatiotemporal variability of neural activity and brain state. The spatial reorganization induced by cognitive activity has been demonstrated using functional connectivity, effective connectivity, and so on [37,54]. Besides those indices, the SSE is effective in revealing state-related reorganization of inter-regional relationship.

Compared with temporal variability, SSE could assess the spatial configuration of neural activity at each time point. The SSE was lower in the task state whereas the temporal variability is greater for better task performance, suggesting that the temporal and spatial dimensions of brain signal variability could reflect different aspects of information during cognition. Therefore, spatial variability and temporal variability could provide complementary information for describing spatiotemporal characteristics of neural activity. In other words, we could get a comprehensive understanding of the matrix of m spatial locations × n time points of the neural signal by combining spatial variability and temporal variability.

Although the reliability and robustness of SSE as a proxy of brain state have been demonstrated, some limitations remain. First, a more important question coming up in the current study and needing further investigation is whether SSE has the power to distinguish different cognitive processes. Second, EEG-based temporal SE and fMRI-based temporal SE reveal signal complexity at different temporal scales. Likewise, voxel-level or region of interest or network analyses may provide valuable information about cognition at different spatial scales, which warrants more investigations.

In summary, we introduced the SE to measure the spatial pattern of the brain in different brain states for the first time. We illuminated that face detection reorganizes the brain into a stable system which consists of predictable brain signal and stable behavioral performance. Combining spatial variability and temporal variability may provide a comprehensive understanding of the spatiotemporal characteristics of neural activity under various brain states.

## References

[1] Garrett DD, Kovacevic N, McIntosh AR, Grady CL. The modulation of BOLD variability between cognitive states varies by age and processing speed. Cerebral Cortex. 2013;23(3):684–93. 10.1093/cercor/bhs055 22419679 PMC3823571

[2] Garrett DD, McIntosh AR, Grady CL. Brain signal variability is parametrically modifiable. Cerebral Cortex. 2014;24(11):2931–40. 10.1093/cercor/bht150 23749875 PMC4193462

[3] Takahashi T. Complexity of spontaneous brain activity in mental disorders. Progress in Neuro-Psychopharmacology & Biological Psychiatry. 2013;45(3):258–66. 10.1016/j.pnpbp.2012.05.001 22579532

[4] Vanessa S, Mazerolle EL, Fisk JD, Ritchie LJ, Gawryluk JR. Resting state BOLD variability in Alzheimer's disease: A marker of cognitive decline or cerebrovascular status? Frontiers in Aging Neuroscience. 2018;10:39 10.3389/fnagi.2018.00039 29515434 PMC5826397

[5] Guitart-Masip M, Salami A, Garrett D, Rieckmann A, Lindenberger U, Bäckman L. BOLD variability is related to dopaminergic neurotransmission and cognitive aging. Cerebral Cortex. 2016;26(5):2074–83. 10.1093/cercor/bhv029 25750252

[6] Boylan MA, Foster CM, Pongpipat EE, E.Webb C, Rodrigue KM, Kennedy KM. Greater BOLD variability is associated with poorer cognitive function in an adult lifespan sample. Cerebral Cortex. 2020;bhaa243:1–13. 10.1093/cercor/bhz066 32915200 PMC7727366

[7] Garrett DD, Samanez-Larkin GR, MacDonald SW, Lindenberger U, McIntosh AR, Grady CL. Moment-to-moment brain signal variability: A next frontier in human brain mapping? Neuroscience & Biobehavioral Reviews. 2013;37(4):610–24. 10.1016/j.neubiorev.2013.02.015 23458776 PMC3732213

[8] Deco G, Kringelbach ML. Metastability and Coherence: Extending the Communication through Coherence Hypothesis Using A Whole-Brain Computational Perspective. Trends in Neurosciences. 2016;39(3):125–35. 10.1016/j.tins.2016.01.001 26833259

[9] Nichols ALA, Eichler T, Latham R, Zimmer M. A global brain state underlies C. elegans sleep behavior. Science. 2017;356(6344):1–9. 10.1126/science.aam6851 28642382

[10] Zuo X-N, Di Martino A, Kelly C, Shehzad ZE, Gee DG, Klein DF, et al The oscillating brain: complex and reliable. NeuroImage. 2010;49(2):1432–45. 10.1016/j.neuroimage.2009.09.037 19782143 PMC2856476

[11] Raichle ME. The brain's default mode network. Annual Review of Neuroscience. 2015;38:433–47. 10.1146/annurev-neuro-071013-014030 25938726

[12] Wang Y, Chen W, Ye L, Biswal BB, Yang X, Zou Q, et al Multiscale energy reallocation during low-frequency steady-state brain response. Human Brain Mapping. 2018;39:2121–32. 29389047 10.1002/hbm.23992 PMC6866265

[13] Zhang H, Zhang L, Zang Y. Fluctuation amplitude and local synchronization of brain activity in the ultra-low frequency band: an fMRI investigation of continuous feedback of finger force. Brain Research. 2015;1629:104–12. 26499258 10.1016/j.brainres.2015.10.023

[14] Xue G, Dong Q, Chen C, Lu Z, Mumford JA, Poldrack RA. Greater Neural Pattern Similarity Across Repetitions Is Associated with Better Memory. Science. 2010;330(6000):97–101. 10.1126/science.1193125 20829453 PMC2952039

[15] Thavabalasingam S, O'Neil EB, Lee A. Multivoxel pattern similarity suggests the integration of temporal duration in hippocampal event sequence representations. Neuroimage. 2018;178:136–46. 10.1016/j.neuroimage.2018.05.036 29775662

[16] Xia T, Zhuang L, Xu X, Qi Z, Luo W. How do different emotional states represent in human brain?——Evidence from multi-variate pattern analysis based on functional MRI. Chinese Science Bulletin. 2018;63(3):241–7.

[17] Costa M, Goldberger AL, Peng CK. Multiscale entropy analysis of complex physiologic time series. Physical Review Letters. 2002;92(8):705–8. 10.1103/PhysRevLett.89.068102 12190613

[18] Courtiol J, Perdikis D, Petkoski S, Müller V, Huys R, Sleimen-Malkoun R, et al The Multiscale Entropy: guidelines for use and interpretation in brain signal analysis. Journal of Neuroscience Methods. 2016;273:175–90. 10.1016/j.jneumeth.2016.09.004 27639660

[19] Gao J, Hu J, Liu F, Cao Y. Multiscale entropy analysis of biological signals: a fundamental bi-scaling law. Frontiers in Computational Neuroscience. 2015;9:64 10.3389/fncom.2015.00064 26082711 PMC4451367

[20] Torre-Luque ADL, Bornas X, Balle M, Fiol-Veny A. Complexity and nonlinear biomarkers in emotional disorders: A meta-analytic study. Neuroscience & Biobehavioral Reviews. 2016;68:410–22. 10.1016/j.neubiorev.2016.05.023 27267791

[21] Goldberger AL, Peng CK, Lipsitz LA. What is physiologic complexity and how does it change with aging and disease? Neurobiology of Aging. 2002;23(1):23–6. 10.1016/s0197-4580(01)00266-4 11755014

[22] Garrett DD, Kovacevic N, McIntosh AR, Grady CL. The importance of being variable. The Journal of Neuroscience. 2011;31(12):4496–503. 10.1523/JNEUROSCI.5641-10.2011 21430150 PMC3104038

[23] Sokunbi MO. Sample entropy reveals high discriminative power between young and elderly adults in short fMRI data sets. Frontiers in Neuroinformatics. 2014;8:69 10.3389/fninf.2014.00069 25100988 PMC4107942

[24] Grandy TH, Garrett DD, Florian S, Markus WB. On the estimation of brain signal entropy from sparse neuroimaging data. Scientific Reports. 2016;6:23073 10.1038/srep23073 27020961 PMC4810375

[25] Mcdonough IM, Nashiro K. Network complexity as a measure of information processing across resting-state networks: evidence from the Human Connectome Project. Frontiers in Human Neuroscience. 2014;8:409 10.3389/fnhum.2014.00409 24959130 PMC4051265

[26] Yang AC, Huang CC, Yeh HL, Liu ME, Hong CJ, Tu PC, et al Complexity of spontaneous BOLD activity in default mode network is correlated with cognitive function in normal male elderly: a multiscale entropy analysis. Neurobiology of Aging. 2013;34(2):428–38. 10.1016/j.neurobiolaging.2012.05.004 22683008

[27] Wang Y-F, Liu F, Long Z-L, Duan X-J, Cui Q, Yan JH, et al Steady-state BOLD response modulates low frequency neural oscillations. Scientific Reports. 2014;4:7376 10.1038/srep07376 25488025 PMC4260215

[28] Lewis LD, Setsompop K, Rosen BR, Polimeni JR. Fast fMRI can detect oscillatory neural activity in humans. Proceedings of the National Academy of Sciences. 2016;113(43):e6679–e85. 10.1073/pnas.1608117113 27729529 PMC5087037

[29] Wang Y-F, Dai G-S, Liu F, Long Z-L, Yan JH, Chen H-F. Steady-state BOLD response to higher-order cognition modulates low frequency neural oscillations. Journal of Cognitive Neuroscience. 2015;27(12):2406–15. 10.1162/jocn_a_00864 26284992

[30] Gao X, Gentile F, Rossion B. Fast periodic stimulation (FPS): a highly effective approach in fMRI brain mapping. Brain Structure and Function. 2018;223(5):2433–54. 10.1007/s00429-018-1630-4 29502144

[31] Wang Y, Liu F, Jing X, Long Z, Chen H. Phase-dependent alteration of functional connectivity density during face recognition in the infra-slow frequency range. In: Wang R, Pan X, editors. Advances in Cognitive Neurodynamics (V). 5. Singapore: Springer Singapore; 2016 p. 305–10.

[32] Wang YF, Long Z, Cui Q, Liu F, Jing XJ, Chen H, et al Low frequency steady‐state brain responses modulate large scale functional networks in a frequency‐specific means. Human Brain Mapping. 2016;37:381–94. 10.1002/hbm.23037 26512872 PMC6867441

[33] Nogueira R, Lawrie S, Moreno-Bote R. Neuronal Variability as a Proxy for Network State. Trends in Neurosciences. 2018;41(4):170–3. 10.1016/j.tins.2018.02.003 29602335

[34] Christoph H, Andreea L, Bernhard N, Jochen T. Where’s the Noise? Key Features of Spontaneous Activity and Neural Variability Arise through Learning in a Deterministic Network. Plos Computational Biology. 2015;11(12):e1004640 10.1371/journal.pcbi.1004640 26714277 PMC4694925

[35] Perdikis D, Huys R, Jirsa VK. Time scale hierarchies in the functional organization of complex behaviors. Plos Computational Biology. 2011;7(9):e1002198 10.1371/journal.pcbi.1002198 21980278 PMC3182871

[36] Churchland MM, Yu BM, Cunningham JP, Sugrue LP, Cohen MR, Corrado GS, et al Stimulus onset quenches neural variability: a widespread cortical phenomenon. Nature Neuroscience. 2010;13(3):369–78. 10.1038/nn.2501 20173745 PMC2828350

[37] Ponce-Alvarez A, He BJ, Hagmann P, Deco G. Task-driven activity reduces the cortical activity space of the brain: experiment and whole-brain modeling. PLoS Computational Biology. 2015;11(8):e100445 10.1371/journal.pcbi.1004445 26317432 PMC4552873

[38] Yan C-G, Zang Y-F. DPARSF: a MATLAB toolbox for “pipeline” data analysis of resting-state fMRI. Frontiers in Systems Neuroscience. 2010;4(13):1–7. 20577591 10.3389/fnsys.2010.00013 PMC2889691

[39] Friston KJ, Williams S, Howard R, Frackowiak RS, Turner R. Movement-related effects in fMRI time-series. Magnetic Resonance in Medicine. 1996;35(3):346–55. 10.1002/mrm.1910350312 8699946

[40] Wang Y, Huang X, Yang X, Yang Q, Wang X, Northoff G, et al Low frequency phase-locking of brain signals contribute to efficient face recognition. Neuroscience. 2019;422:172–83. 10.1016/j.neuroscience.2019.10.024 31704494

[41] Lu FM, Wang YF, Zhang J, Chen HF, Yuan Z. Optical mapping of the dominant frequency of brain signal oscillations in motor systems. Scientific Reports. 2017;7:14703 10.1038/s41598-017-15046-9 29116158 PMC5677051

[42] Fan L, Li H, Zhuo J, Zhang Y, Wang J, Chen L, et al The Human Brainnetome Atlas: A New Brain Atlas Based on Connectional Architecture. Cerebral Cortex. 2016;26(8):3508–26. 10.1093/cercor/bhw157 27230218 PMC4961028

[43] Wang Y, Wang X, Ye L, Yang Q, Cui Q, He Z, et al Spatial complexity of brain signal is altered in patients with generalized anxiety disorder. Journal of Affective Disorders. 2019;246:387–93. 10.1016/j.jad.2018.12.107 30597300

[44] DeSalvo MN, Douw L, Takaya S, Liu H, Stufflebeam SM. Task‐dependent reorganization of functional connectivity networks during visual semantic decision making. Brain and Behavior. 2014;4(6):877–85. 10.1002/brb3.286 25365802 PMC4178300

[45] Grady CL, Garrett DD. Brain signal variability is modulated as a function of internal and external demand in younger and older adults. NeuroImage. 2018;169:510–23. 10.1016/j.neuroimage.2017.12.031 29253658

[46] Binder JR. Task-induced deactivation and the "resting" state. Neuroimage. 2012;62(2):1086–91. 10.1016/j.neuroimage.2011.09.026 21979380 PMC3389183

[47] Newell KM, Broderick MP, Deutsch KM, Slifkin AB. Task goals and change in dynamical degrees of freedom with motor learning. Journal of Experimental Psychology Human Perception & Performance. 2003;29(2):379–87. 10.1037/0096-1523.29.2.379 12760622

[48] Vaillancourt DE, Newell KM. Changing complexity in human behavior and physiology through aging and disease. Neurobiology of Aging. 2002;23(1):1–11. 10.1016/s0197-4580(01)00247-0 11755010

[49] Iaccarino HF, Singer AC, Martorell AJ, Rudenko A, Gao F, Gillingham TZ, et al Gamma frequency entrainment attenuates amyloid load and modifies microglia. Nature. 2016;540(7632):230–5. 10.1038/nature20587 27929004 PMC5656389

[50] Wang Y, Zhu L, Zou Q, Cui Q, Liao W, Duan X, et al Frequency dependent hub role of the dorsal and ventral right anterior insula. Neuroimage. 2018;165:112–7. 10.1016/j.neuroimage.2017.10.004 28986206

[51] Li L, Wang Y, Ye L, Chen W, Huang X, Cui Q, et al Altered brain signal variability in patients with generalized anxiety disorder. Frontiers in Psychiatry. 2019;10:84 10.3389/fpsyt.2019.00084 30886589 PMC6409298

[52] Zhang J, Cheng W, Liu Z, Zhang K, Lei X, Yao Y, et al Neural, electrophysiological and anatomical basis of brain-network variability and its characteristic changes in mental disorders. Brain. 2016;139(8):2307–21. 10.1093/brain/aww143 27421791

[53] Dinstein I, Heeger DJ, Behrmann M. Neural variability: friend or foe? Trends in Cognitive Sciences. 2015;19(6):322–8. 10.1016/j.tics.2015.04.005 25979849

[54] Di X, Biswal BB. Toward Task Connectomics: Examining Whole-Brain Task Modulated Connectivity in Different Task Domains. Cerebral Cortex. 2019;29(4):1572–83. 10.1093/cercor/bhy055 29931116 PMC7302740

